# Supplementary material for: Causal Relationship between Aging and Anorexia Nervosa: A White-Matter-Microstructure-Mediated Mendelian Randomization Analysis
Source: Biomedicines. 2024 Aug 16;12(8):1874. doi: 10.3390/biomedicines12081874 (PMC11351342; doi:10.3390/biomedicines12081874)
Supplement: Supplementary file 1 [file biomedicines-12-01874-s001.zip › Supplementary Figure/Figure S4/Singe SNP plot of MR analysis for TL to ubm-b-1999.pdf]

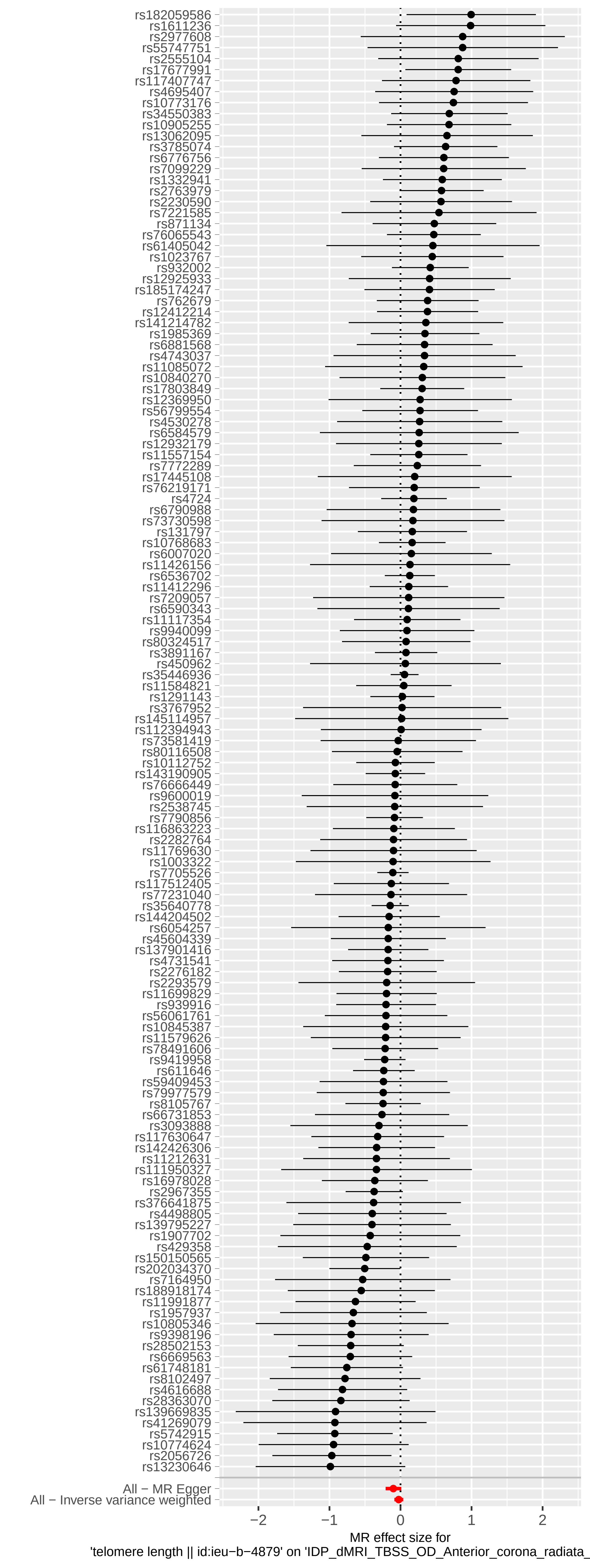
rs182059586
rs1611236
rs2977608
rs55747751
rs2555104
rs17677991
rs117407747
rs4695407
rs10773176
rs34550383
rs10905255
rs13062095
rs3785074
rs6776756
rs7099229
rs1332941
rs2763979
rs2230590
rs7221585
rs871134
rs76065543
rs61405042
rs1023767
rs932002
rs12925933
rs185174247
rs762679
rs12412214
rs141214782
rs1985369
rs6881568
rs4743037
rs11085072
rs10840270
rs17803849
rs12369950
rs56799554
rs4530278
rs6584579
rs12932179
rs11557154
rs7772289
rs17445108
rs76219171
rs4724
rs6790988
rs73730598
rs131797
rs10768683
rs6007020
rs11426156
rs6536702
rs11412296
rs7209057
rs6590343
rs11117354
rs9940099
rs80324517
rs3891167
rs450962
rs35446936
rs11584821
rs1291143
rs3767952
rs145114957
rs112394943
rs73581419
rs80116508
rs10112752
rs143190905
rs76666449
rs9600019
rs2538745
rs7790856
rs116863223
rs2282764
rs11769630
rs1003322
rs7705526
rs117512405
rs77231040
rs35640778
rs144204502
rs6054257
rs45604339
rs137901416
rs4731541
rs2276182
rs2293579
rs11699829
rs939916
rs56061761
rs10845387
rs11579626
rs78491606
rs9419958
rs611646
rs59409453
rs79977579
rs8105767
rs66731853
rs3093888
rs117630647
rs142426306
rs11212631
rs111950327
rs16978028
rs2967355
rs376641875
rs4498805
rs139795227
rs1907702
rs429358
rs150150565
rs202034370
rs7164950
rs188918174
rs11991877
rs1957937
rs10805346
rs9398196
rs28502153
rs6669563
rs61748181
rs8102497
rs4616688
rs28363070
rs139669835
rs41269079
rs5742915
rs10774624
rs2056726
rs13230646
All − MR Egger
All − Inverse variance weighted
−2
−1
0
1
2
MR effect size for
'telomere length || id:ieu−b−4879' on 'IDP_dMRI_TBSS_OD_Anterior_corona_radiata_
